# High‐grade medial femoral condyle and medial tibial plateau bone bruises predict ramp lesions of the medial meniscus in patients with anterior cruciate ligament tears: A prospective clinical and MRI evaluation

**DOI:** 10.1002/jeo2.70238

**Published:** 2025-04-13

**Authors:** Filippo Familiari, Luke V. Tollefson, Antonio Izzo, Raffaella Russo, Michele Mercurio, Giorgio Gasparini, Robert F. LaPrade, Giovanni Di Vico

**Affiliations:** ^1^ Department of Orthopaedic and Trauma Surgery Magna Graecia University Catanzaro Italy; ^2^ Research Center on Musculoskeletal Health, MusculoSkeletal Health@UMG Magna Graecia University Catanzaro Italy; ^3^ Twin Cities Orthopedics Edina Minnesota USA; ^4^ Department of Public Health, Trauma and Orthopaedics University Federico II Napoli Italy; ^5^ Casa di Cura San Michele Maddaloni Italy; ^6^ Division of Nutrition Clinic, Department of Medical and Surgical Sciences Magna Graecia University Catanzaro Italy

**Keywords:** anterior cruciate ligament, bone bruise, medial meniscus ramp tear

## Abstract

**Purpose:**

The purpose of this study was to evaluate potential predictive diagnostic variables on magnetic resonance imaging (MRI) for medial meniscus ramp tears in the presence of an anterior cruciate ligament (ACL) tear.

**Methods:**

Patients aged ≥16 years undergoing arthroscopic anatomic hamstring single‐bundle ACL reconstruction between July 2016 and September 2020 from a single centre were prospectively enroled with a diagnosis based on clinical and MRI evaluations. Demographic data such as age, gender, dominant limb and time from injury to surgery were recorded. Clinical assessments included Lachman and pivot shift tests. Imaging assessments involved grading bone bruises on MRI using the International Cartilage Repair Society (ICRS) scale. Statistical analysis was conducted using parametric tests and regression analysis with a *p* value of less than 0.05, which is considered significant.

**Results:**

The final sample consisted of 108 patients, with a concomitant ACL tear and medial meniscus ramp tear present in 53 (49.1%) patients. In the univariate regression analysis, a higher grade of the medial femoral condyle and medial tibial plateau bone bruises was highly associated with the diagnosis of an ACL tear with a concomitant ramp tear (*p* = 0.006, *β* = 0.151 and *p* < 0.001, *β* = 0.172, respectively). In the univariate regression analysis, a higher grade of the medial femoral condyle and medial tibial plateau bone bruise was associated with the diagnosis of ACL tear with a concomitant ramp tear (*p* = 0.006, *β* = 0.151 and *p* < 0.001, *β* = 0.172, respectively).

**Conclusion:**

In this prospective series of patients with ACL tears, 49% were found to have concomitant medial meniscal ramp tears. The finding of medial tibial plateau or medial femoral condyle bone bruising was predictive of ramp tear. The presence of this bone bruising pattern along with a high‐grade Lachman and/or pivot shift examination, a medial meniscus ramp tear should be suspected.

**Level of Evidence:**

Level II, prospective cohort study.

**Abbreviations**
ACL: anterior cruciate ligament
ACLR: anterior cruciate ligament reconstruction
BMI: body mass index
CI: confidence interval
DICOM: Digital Imaging and Communications in Medicine
ICRS: International Cartilage Repair Society
LCL: lateral collateral ligament
MCL: medial collateral ligament
MRI: magnetic resonance imaging
PCL: posterior cruciate ligament
SD: standard deviation
SPSS: Statistical Package for the Social Sciences
ST: semitendinosus

## INTRODUCTION

Medial meniscus ramp tears commonly occur in conjunction with tears of the anterior cruciate ligament (ACL) [10]. Medial meniscus ramp tears are longitudinal tears of the capsule at the posterior horn of the medial meniscus [2]. These tears commonly affect the meniscocapsular (femoral‐sided) or meniscotibial (tibial‐sided) attachments of the capsule to the meniscus [2, 18]. Previous studies have highlighted the anatomy of the ramp attachment reporting that the meniscocapsular and meniscotibial attachments form together in a common attachment site at the posterior medial meniscus [18].

Tears of the medial meniscus ramp are often called ‘hidden lesions’ due to the difficulty in identifying the tear with standard anterior arthroscopic portals. In many cases, these tears must be diagnosed with a trans‐notch view of the posteromedial compartment [10]. This is due to the meniscocapsular attachment to the medial meniscus, which occurs at a depth of 36% of the total meniscal height [18]. As the recognition and awareness of ramp tears increase, their reported incidence is also increasing [9, 10]; the incidence of ACL tears with medial meniscus ramp tears has recently been reported to be between 22.9% and 40.8% [12, 22, 28].

Medial meniscus ramp tears have detrimental effects on overall knee stability [11]. Biomechanical studies have reported that concomitant ACL and medial meniscus ramp tears increase anterior tibial translation and internal rotation of the knee compared to an isolated ACL tear state [2, 17, 27]. Repairing a ramp tear can restore near‐native knee biomechanics compared to the unrepaired state [17, 27]. Clinically, patients have reported improved post‐operative patient‐reported outcomes after undergoing ACL reconstruction (ACLR) with a medial meniscus ramp repair [1, 13, 15].

Various studies have attempted to improve the diagnosis of medial meniscus ramp tears due to the low sensitivity of a diagnosis and the difficulty of an arthroscopic diagnosis. Current literature reports on the sensitivity of the MRI diagnosis of ramp tears between 48% and 68%, emphasising the need for proper arthroscopic evaluation [14, 31]. Current risk factors for concomitant ACL and medial meniscus ramp tears include contact sports, high‐grade pivot shift with lateral femoral condyle and posterolateral tibial plateau bone bruise, increased posterior tibial slope and Segond fractures [3, 12].

The purpose of this study was to evaluate predictive diagnostic variables on MRI scans to determine if any are associated with the presence or absence of a ramp tear with a concomitant ACL tear. Our null hypothesis was that there would be no significant differences between the cohorts of isolated ACLR and ACLR with ramp lesions.

## MATERIALS AND METHODS

The local institutional review board approved the study protocol, and the research was conducted in compliance with the Declaration of Helsinki. Patients undergoing primary unilateral arthroscopic anatomic single‐bundle autograft hamstring ACL reconstruction from a single centre between July 2016 and September 2020 were prospectively enroled as the study group, and informed consent was obtained.

### Patient involvement statement

Eligibility criteria were as follows: (1) ≥16 years old at the time of surgery, (2) ACLR using semitendinosus and gracilis tendon autograft (ST‐G), and (3) the capability to communicate with healthcare professionals and provide informed consent. Exclusion criteria included previous surgeries of the affected knee, previous or concomitant lesions of the posterior cruciate ligament (PCL) and of the medial and lateral collateral ligaments (MCL and LCL) and previous fractures of the ipsilateral femur and tibia as well as patients demonstrating severe knee osteoarthritis (Kellgren–Lawrence Grades III and IV) [25, 26]. Patients who had suffered a direct contact injury, as this could provoke other bone bruises, and patients who could not undergo an MRI within 6 weeks of the injury event were also excluded. The mean time elapsed between the injury event and the MRI was 18 ± 5 days (range: 10–40 days).

The diagnosis of ACL tear was based on clinical and MRI evaluation of the knee (within 6 weeks of the ACL tear). A total of 108 patients met the study criteria. Demographic data, including age, gender, dominant limb defined as the preferred kicking limb and the injury to surgery time interval, were assessed [20]. The clinical assessment of patients included performing the Lachman and pivot shift exams.

### Imaging assessment

Magnetic resonance imaging (MRI) scans of the involved knee were available for all patients evaluated. All imaging measurements were carried out using a DICOM medical image viewer (Horos Project; Purview).

**Figure 1:**
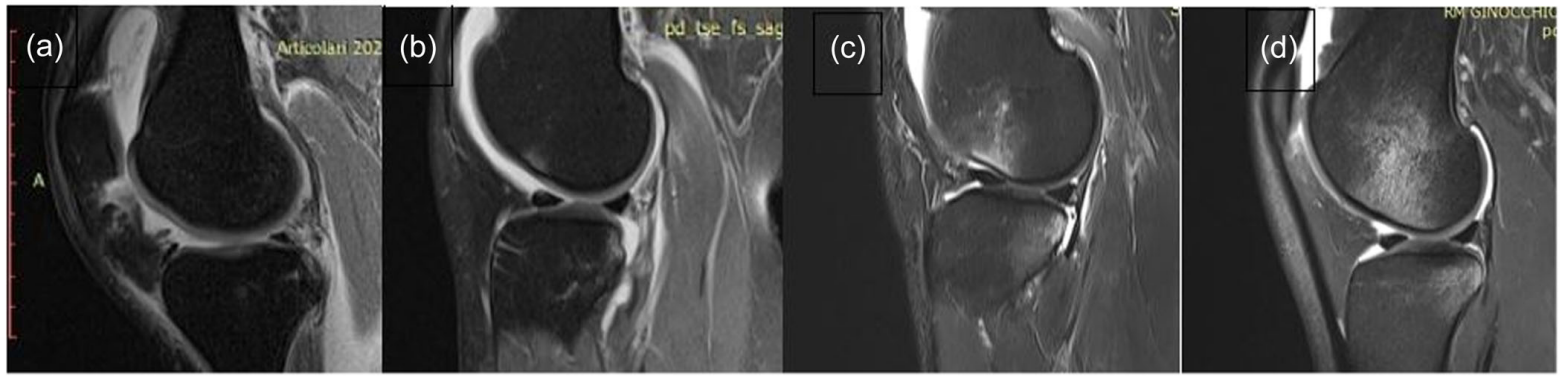
Classification of bone bruise into four grades for medial femoral condyle on magnetic resonance imaging according to the ICRS scale: a, absent; b, minimal; c, moderate; and d, severe [8]. ICRS, International Cartilage Repair Society.

**Figure 2:**
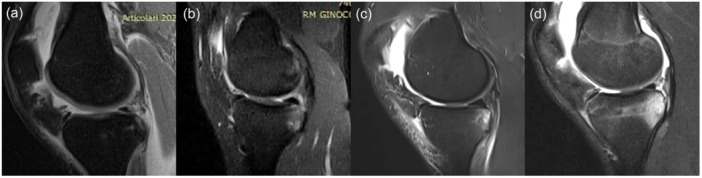
Classification of bone bruising into four grades for medial tibial plateau bone bruising on magnetic resonance imaging according to the ICRS scale: a, absent; b, minimal; c, moderate; and d, severe. ICRS, International Cartilage Repair Society.

The presence and extent of bone bruises at the medial femoral condyle and at the medial tibial plateau were determined on 1.5 T unit MRI scans (Achieva XR, Philips Medical System) on PD‐FS sequences and classified into one of the four grades on the International Cartilage Repair Society (ICRS) scale described by Brittberg and Winalski [8] analyzed as follows: Grade 0, absent; Grade 1, minimal (only at subchondral bone); Grade 2, moderate (extension from articular surface to growth plate) and Grade 3, severe (extension beyond growth plate)— (Figures 1 and 2).

All measurements were performed by two independent surgeons (A.I. and R.R.) blinded with the aim of the study to assess the interobserver reliability. The same surgeons performed the same measurements after 4 weeks to assess the intra‐observer reliability [29]. During arthroscopy for all patients with an ACL tear, ramp lesions were identified by probing the meniscus with anterior and trans‐notch (Gillquist manoeuvre) visualization. A valgus force on the knee can aid in opening the medial compartment for better visualization of the medial meniscus. Any other meniscus tears, either in the medial or lateral menisci, were assessed and recorded.

### Statistical analysis

All data were collected, measured, and reported at an accuracy of one decimal place. Continuous variables are presented as the mean, standard deviation (SD), and range, and categorical variables are presented as counts. For comparison, two cohorts of patients were established, one with isolated ACL tears and the other with ACL tears and concomitant medial meniscus ramp tears. The distribution of the numeric samples was assessed using the Kolmogorov‒Smirnov normality test. Based on this preliminary analysis, parametric tests were adopted. Unpaired Student's *t* tests were used to analyze the significance of the differences. The differences for categorical variables were tested by *χ*2 (chi‐squared) test.

Regression analysis was performed on the entire patient population to test for potential predictors for medial meniscus ramp tears. The explanatory and confounding pre‐ and post‐operative variables included in the analysis were sex (categorical), age (continuous), dominant limb (categorical), body mass index (BMI) (continuous) and concomitant meniscal lesion (categorical). Only explanatory and confounding variables that showed a trend toward an association (e.g., *p* < 0.10) with the outcome of interest in the univariate analysis were included in the multiple regression analysis. Cohen's kappa coefficient was adopted to measure the reliability of MRI assessments of bone bruising. IBM SPSS Statistics software (version 26, IBM Corp.) and G*Power (version 3.1.9.2, Institut für Experimentelle Psychologie, Heinrich Heine Universität) were used to construct the database and perform statistical analysis. A *p* value of less than 0.05 was considered significant.

A prior sample size calculation was performed considering previous studies (G*Power, version 3.1.9.2, Institut fur Experimentelle Psychologie, Heinrich Heine Universitat) [29, 30]. A minimum of 50 patients per group was determined to satisfy a medium–large effect size with 80% power and statistical significance at an alpha level of 0.05.

## RESULTS

The demographic and MRI characteristics of the included patients are summarized in Table 1. The final sample consisted of 108 patients, 102 (94.4%) of whom were male, with an average age of 26.9 ± 8 years (range: 16–50 years) at surgery.

**Table 1:** Baseline characteristics of included patients.

Patients (No. = 108)	Mean ± SD (range) or No. (%)
Gender	
Male	102 (94.4%)
Female	6 (5.6%)
Age at surgery (years)	26.9 ± 8 (16–50)
BMI	24 ± 4.2 (21–27)
Dominant limb	90 (83.3%)
Injury to surgery interval (months)	5.6 ± 7.6 (1–60)
Medial femoral condyle bone bruise	
Grade 0	39 (36.1%)
Grade 1	40 (37%)
Grade 2	24 (22.2%)
Grade 3	5 (4.6%)
Mean grade	1 ± 0.9 (0–3)
Medial tibial plateau bone bruise	
Grade 0	30 (27.8%)
Grade 1	42 (38.9%)
Grade 2	27 (25%)
Grade 3	9 (8.3%)
Mean grade	1.1 ± 0.9 (0–3)
Concomitant meniscal lesion	
Anterior horn medial meniscus tear	18 (16.7%)
Ramp tear	53 (49.1%)
Lateral meniscus tear	20 (18.5%)
Concomitant cartilage defects (Grades I and II ICRS)	4 (3.7%)

Abbreviations: BMI, body mass index; ICRS, International Cartilage Repair Society; No., number of cases; SD, standard deviation.

A concomitant ACL tear and medial meniscus ramp tear were present in 53 (49.1%) patients. Overall, a concomitant tear of the anterior horn of the medial meniscus (outside the zone of a meniscus ramp tear area) and of the lateral meniscus was present in 18 (16.7%) and 20 (18.5%) patients, respectively.

All demographic and MRI factors were compared between isolated ACL and ACL with concomitant ramp lesion groups to assess for associations (Table 2).

**Table 2:** Differences between the isolated ACL and ACL with concomitant ramp lesion groups.

	ACL group	ACL and ramp lesion group	*p*	95% CI	SED
	Mean ± SD (range) or No. (%)	Mean ± SD (range) or No. (%)			
Gender					
Male	51 (92.7%)	51 (96.2%)	0.679		
Age at surgery (years)	25.6 ± 8.1 (16–50)	28.2 ± 7.8 (16–48)	0.095		
BMI	24 ± 2 (21–27)	24.3 ± 1.3 (21–27)	0.321		
Dominant limb	49 (89.1%)	41 (77.4%)	0.125		
Injury to surgery interval (months)	4.6 ± 4.7 (1–36)	6.8 ± 9.8 (1–60)	0.195		
Medial femoral condyle bone bruise					
Grade 0	24 (36.1%)	15 (28.3%)	0.112		
Grade 1	23 (37%)	17 (32.1%)	0.324		
Grade 2	7 (22.2%)	17 (32.1%)	**0.021**		
Grade 3	1 (4.6%)	4 (7.5%)	0.201		
Mean grade	0.7 ± 0.8 (0–3)	1.2 ± 0.9 (0–3)	**0.006**	**−0.79 to −0.14**	**0.164**
Medial tibial plateau bone bruise					
Grade 0	22 (27.8%)	8 (15.1%)	**0.005**		
Grade 1	20 (38.9%)	22 (41.5%)	0.694		
Grade 2	12 (25%)	15 (28.3%)	0.508		
Grade 3	1 (8.3%)	8 (15.1%)	**0.015**		
Mean grade	0.9 ± 0.8 (0–3)	1.4 ± 0.9 (0–3)	**<0.001**	**−0.91 to −0.24**	**0.169**
Concomitant meniscal lesion					
Anterior horn medial meniscus tear	8 (14.5%)	10 (18.9%)	0.612		
Lateral meniscus tear	7 (12.7%)	13 (24.5%)	0.141		
Concomitant cartilage defects (Grades I and II ICRS)	0 (0%)	4 (7.5%)	0.055		

*Note*: *p* < 0.05 are in bold.

Abbreviations: ACL, anterior cruciate ligament; CI, confidence interval; ICRS, International Cartilage Repair Society; No., number of cases; PCL, posterior cruciate ligament; SD, standard deviation; SED, standard error of difference.

Overall, higher mean grades of the medial femoral condyle and medial tibial plateau bone bruise on MRI were found in the ACL tear with concomitant ramp tear group (*p* = 0.006 and *p* < 0.001, respectively). A higher rate of Grade 2 of the medial femoral condyle bone bruise was found in the ACL tear with a concomitant ramp tear group (*p* = 0.021). A lower rate of Grade 0 and a higher rate of Grade 3 of the medial tibial plateau bone bruise were also found in the ACL tear with concomitant ramp tear group (*p* = 0.005 and *p* = 0.015, respectively). No differences were found in terms of demographic data and concomitant meniscal lesions (other meniscus tears not including ramp tears) between the groups. Cohen's kappa coefficients for intraobserver and interobserver reliability of MRI assessments of bone bruising were 0.88 and 0.86, respectively.

In the regression analysis, a higher grade of the medial femoral condyle and medial tibial plateau bone bruise was associated with the diagnosis of ACL tear with a concomitant ramp tear (*p* = 0.006, *β* = 0.151 and *p* < 0.001, *β* = 0.172, respectively). The multivariate analysis confirmed the association between a higher grade of the medial tibial plateau bone bruise and the diagnosis of ACL tear with a concomitant ramp tear (*p* = 0.001, *β* = 0.519).

## DISCUSSION

The most important finding from this study was that femoral and tibial bone bruises were associated with a medial meniscus ramp tear in patients with an ACL tear. A higher grade of both a medial tibial plateau bone bruise and a medial femoral condyle bone bruise was associated with the diagnosis of an ACL tear with a concomitant ramp tear. These findings suggest that meniscal ramp tears that occur with ACL tears may be suspected on these secondary MRI signs, especially by observing the extent of bone bruise of the tibial side of the knee joint.

The findings from this study help to provide additional tools for diagnosing medial meniscus ramp tears. Ramp tears identification requires careful evaluation of the MRI as well as special attention to probing and direct visualization at the time of arthroscopy. On MRI, the sensitivity of a diagnosis is low, being reported between 48% and 68%, with various authors highlighting the difficulties of diagnosis [7, 10, 14, 31]. Even when there appears to be no ramp tear on MRI, arthroscopic probing should be performed to assess the meniscocapsular and meniscotibial attachment. The meniscocapsular attachment of the medial meniscus is often called the ‘hidden zone’ because it attaches 36% inferiorly to the superior margin of the meniscus [18]. Due to this ‘hidden zone’, ramp tears can only be properly identified with a standard anterior portal 60% of the time [32]. Lesions in the ‘hidden zone’ must be visualized with a trans notch view and thoroughly probed to identify the extent of the tear [16]. Although medial tibial plateau and medial femoral condyle bone bruising were not present in all cases with a medial meniscus ramp tear, when they are present, a ramp tear should be suspected, and thorough probing should be performed. Other diagnostic tools for assessing the presence of a medial meniscus ramp tear include high‐grade Lachman and pivot shift exams and increased posterior tibial slope [15, 19, 24]. All these tools, including bone bruising on the medial femoral condyle and medial tibial plateau, should be utilized for the workup of a patient with an ACL tear to determine the status of the ramp attachment.

Bone bruises after an injury are common in the knee with various bone bruise morphologies being associated with distinct injury pathologies. For ramp tears specifically, a previous study by Moran et al. [30] reported the association between medial tibial plateau bone bruises and ramp tears for patients with posterolateral corner injuries. This study adds to these findings by reporting on patients with ACL tears and no concomitant ligament pathology and by reporting on the presence of medial femoral condyle bone bruising. Furthermore, bone bruising has been described in detail for patients with tears of different knee ligaments. Studies by Bernholt et al. [4, 5, 6] have reported the presence of posterolateral tibial plateau and lateral femoral condyle bone bruising in patients with ACL tears. Other studies have reported hyperextension‐type bone bruising for posterolateral corner injuries, medial compartment bone bruising for posterior lateral corner injuries, and lateral compartment bone bruising for MCL injuries [21, 23]. The findings from this study aim to provide further explanation for the presence of medial tibial plateau and medial femoral condyle bone bruising in the setting of ACL tears and provide another diagnostic tool in the assessment of ramp tears.

Several limitations are present in this study. One potential limitation was the limited sample size; a larger sample size would help increase statistical power. Another limitation was that most of the patients with ACL tears in this study were males, so further studies to assess secondary signs of meniscal ramp tears in females would be indicated. Future research on this topic is indicated because the peer‐reviewed literature on this subject is limited.

## CONCLUSION

In this prospective series of patients with ACL tears, 49% were found to have concomitant medial meniscal ramp tears. The finding of medial tibial plateau or medial femoral condyle bone bruising was predictive of ramp tear. The presence of this bone bruising pattern along with a high‐grade Lachman and/or pivot shift examination, a medial meniscus ramp tear should be suspected.

## AUTHOR CONTRIBUTIONS

Filippo Familiari, Giovanni Di Vico, Luke V. Tollefson and Antonio Izzo contributed to the study design, data collection and manuscript preparation. Raffaella Russo and Michele Mercurio were involved in data analysis and manuscript review. Robert F. LaPrade and Giovanni Di Vico provided clinical input and manuscript editing. Giorgio Gasparini and Robert F. LaPrade supervised the research and approved the final manuscript.

## CONFLICT OF INTEREST STATEMENT

The authors declare no conflicts of interest.

## ETHICS STATEMENT

This study was conducted following the Declaration of Helsinki and approved by the Institutional Review Board: Comitato Etico Territoriale Regione Calabria (132/2024). Informed consent was obtained from all individual participants included in the study.

## Data Availability

The data that support the findings of this study are available upon reasonable request from the corresponding author.
